# Redefining Cancer Research Priorities in Low- and Middle-Income Countries in the Post–COVID-19 Global Context: A Modified Delphi Consensus Process

**DOI:** 10.1200/GO.23.00111

**Published:** 2023-08-10

**Authors:** Louis Fox, Aida Santaolalla, Jasmine Handford, Richard Sullivan, Julie Torode, Verna Vanderpuye, C.S. Pramesh, Layth Mula-Hussain, Shaymaa AlWaheidi, Lydia E. Makaroff, Ranjit Kaur, Clara Mackay, Deborah Mukherji, Mieke Van Hemelrijck

**Affiliations:** ^1^ Translational Oncology and Urology Research (TOUR), Centre for Cancer, Society and Public Health, King's College London, London, United Kingdom; ^2^ Global Oncology Group, Centre for Cancer, Society and Public Health, King's College London, London, United Kingdom; ^3^ National Centre for Radiotherapy, Oncology and Nuclear Medicine, Korle Bu Teaching Hospital, Accra, Ghana; ^4^ Tata Memorial Centre, Homi Bhabha National Institute, Mumbai, India; ^5^ Sultan Qaboos Comprehensive Cancer Care and Research Centre, Muscat, Oman; ^6^ World Bladder Cancer Patient Coalition, Brussels, Belgium; ^7^ Advanced Breast Cancer Global Alliance, Petaling Jaya, Malaysia; ^8^ World Ovarian Cancer Coalition, Toronto, ON, Canada; ^9^ Naef K Basile Cancer Institute, American University of Beirut Medical Center, Beirut, Lebanon

## Abstract

**PURPOSE:**

The post–COVID-19 funding landscape for cancer research globally has become increasingly challenging, particularly in resource-challenged regions (RCRs) lacking strong research ecosystems. We aimed to produce a list of priority areas for cancer research in countries with limited resources, informed by researchers and patients.

**METHODS:**

Cancer experts in lower-resource health care systems (as defined by the World Bank as low- and middle-income countries; N = 151) were contacted to participate in a modified consensus-seeking Delphi survey, comprising two rounds. In round 1, participants (n = 69) rated predetermined areas of potential research priority (ARPs) for importance and suggested missing ARPs. In round 2, the same participants (n = 49) rated an integrated list of predetermined and suggested ARPs from round 1, then undertook a forced choice priority ranking exercise. Composite voting scores (*T*-scores) were used to rank the ARPs. Importance ratings were summarized descriptively. Findings were discussed with international patient advocacy organization representatives.

**RESULTS:**

The top ARP was research into strategies adapting guidelines or treatment strategies in line with available resources (particularly systemic therapy) (*T* = 83). Others included cancer registries (*T =* 62); prevention (*T* = 52); end-of-life care (*T* = 53); and value-based and affordable care (*T* = 51). The top COVID-19/cancer ARP was strategies to incorporate what has been learned during the pandemic that can be maintained posteriorly (*T* = 36). Others included treatment schedule interruption (*T* = 24); cost-effective reduction of COVID-19 morbidity/mortality (*T* = 19); and pandemic preparedness (*T* = 18).

**CONCLUSION:**

Areas of strategic priority favored by cancer researchers in RCRs are related to adaptive treatment guidelines; sustainable implementation of cancer registries; prevention strategies; value-based and affordable cancer care; investments in research capacity building; epidemiologic work on local risk factors for cancer; and combatting inequities of prevention and care access.

## INTRODUCTION

The impact of cancer in resource-challenged regions (RCRs) is expected to increase dramatically over the next 50 years, largely as a result of rising life expectancy in growing populations but also because of lifestyle changes resulting in lack of physical activity, higher intake of high-fat diets, and increasing tobacco and alcohol consumption.^1^ The expected epidemiological transitions are of particular concern in the many countries with limited financial resources that do not have universal health coverage; and where there are low rates of early-stage detection, relative lack of access to high-quality trimodality treatment, and poor treatment outcomes contribute to global inequities in cancer mortality.^2,3^ The factors contributing to geographic deficiencies in cancer control are complex and regionally idiosyncratic (albeit with resource availability a common theme) and these have been widely discussed.^4-6^ However, there is a common view that high-impact strategies to address these deficiencies must be predicated on robust local research at the country and region level.^7-9^

**CONTEXT**

**Key Objective**
To identify important cancer research priorities in regions with lower resource health care systems in the post–COVID-19 context.
**Knowledge Generated**
Ranked lists of general, and COVID-19–related, cancer research priorities are reported, based upon input from a globally representative group of cancer experts working in resource-challenged regions (RCRs), and validated by representatives from global cancer advocacy organizations.
**Relevance**
These priorities, based on the principle of empowering local voices, can be used to form the basis of effective strategies to ensure high-impact cancer research in RCRs, where the negative impact of the COVID-19 pandemic on available funding for cancer research has been particularly acutely felt.

It is against this backdrop that early in 2020, the world experienced a major pandemic—COVID-19—because of the novel SARS-CoV-2 virus. The emergence of this coronavirus created a major public health cataclysm^10^ and challenges to complex health policy.^11^ At the time of its emergence, the lack of available effective treatments or vaccines forced policymakers into implementing varying degrees of nonpharmaceutical measures, including mandatory isolation orders for entire populaces.^12-14^ The economic impacts of such interventions, combined with the mortality and disability caused by the disease, have had severe economic impacts, which were transmitted throughout the globalized economy.^14-18^ These events have had powerfully negative impacts on available funding for cancer research globally.^19-21^ The impacts have been felt across all resource settings, but most acutely felt in RCR settings,^22,23^ as research capacity was already limited before COVID-19.^24^

In this paper, we use the term RCRs to acknowledge the geospatial complexity of the various economic, political, social and/or environmental challenges faced differentially by geographic regions, both within and across national borders. In the context of this study, the term RCR refers to regions which, for whatever reason, face challenges accessing the resources demanded by a robust cancer control infrastructure. In this sense, the term RCR enables a more nuanced discussion of regional health system complexity than the economic (and reductive) term *low- and middle-income countries* (LMICs).

**FIG 1:**
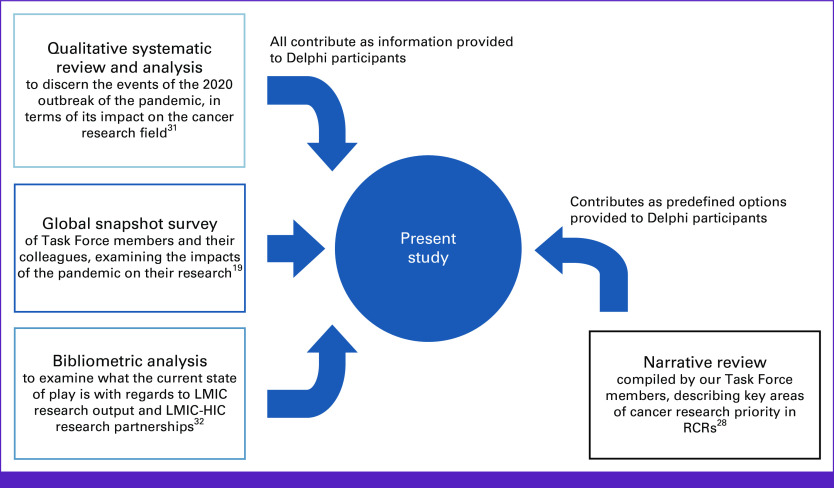
Summary of background work contributing to this study (citations included^19,28,31,32^). The findings of the qualitative systematic review, global snapshot survey, and bibliometric analysis were provided to participants to inform their decisions. The insights from the narrative review were used to formulate predefined areas of potential research priority for participants to scrutinize. HIC, high-income countries; LMIC, low- and middle-income country; RCR, resource-challenged region.

We note that the dynamics of the relationship of global health to RCRs have often been debated, because of a systemic tendency for global health initiatives to prioritize a benefit for individuals and institutions from high-income countries (HICs) over benefit for those in RCRs.^25^ A strong case has been made for approaches that avoid repeating exploitative practices and orient global collaboration toward empowering communities in resource-limited settings, to address local problems with local solutions.^26^ It is in this spirit that our COVID-19 and Cancer Global Task Force^27^ was created in the wake of the COVID-19 pandemic, bringing together clinical and research expertise from RCRs—and some HICs—from around the world to understand how to protect and build upon cancer research activity globally. As part of the Task Force activities, we initiated a project titled Redefining Cancer Research Priorities in Low- and Middle-Income Countries in the Emergent Context of the COVID-19 Pandemic (REPRISE) to (1) analyze the pandemic's impact on the cancer research ecosystem generally; and (2) understand how to build resilience in cancer research ecosystems in RCRs, in this emergent context. The REPRISE project consisted of four work packages, which have all fed into the study presented here (Fig 1).

Building upon this previous work, this study aimed to conduct a modified Delphi consensus-seeking process, to identify the most important research priorities in cancer in RCRs according to a globally sampled group of individuals from RCRs, or with extensive experience in the RCR context. Our objectives were to (1) ask our sample what they think the priorities for cancer research in RCRs should be; and (2) to rank these research priorities according to their relative importance.

## METHODS

### Study Design

We conducted a modified Delphi survey, comprising two rounds, to establish consensus among cancer experts working in RCRs as to what cancer research in RCRs should be prioritized.

**FIG 2:**
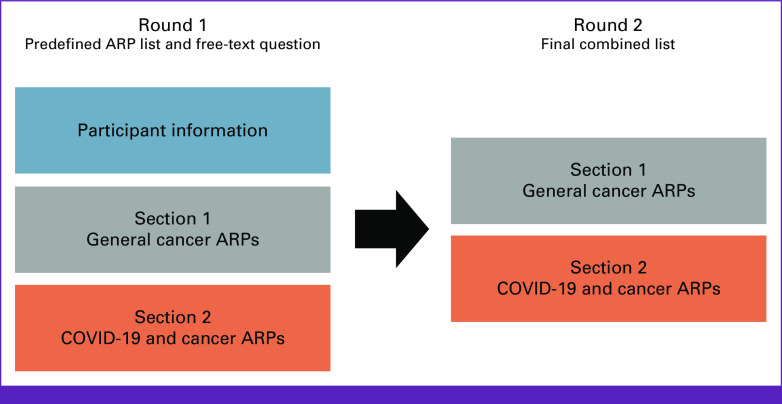
Layout of procedures. ARPs, areas of potential research priority.

In round 1, participants were presented with a predefined list of areas of potential research priority (ARPs), on the basis of previous work conducted by our COVID-19 and Cancer Global Task Force.^28^ In round 2, the same individuals were presented with a combined ARP list resulting from round 1 (ie, both the predefined ARPs and the participant-contributed ARPs). Each round was divided into two sections: (1) *general cancer* ARPs and (2) *COVID-19 and cancer* ARPs (Fig 2).

As recommended by others to enhance rigor in the Delphi process,^29^ the results of this process were presented to three individuals (in three separate interviews) representing international patient advocacy organizations, as a validation process and to obtain qualitative feedback.

This study was granted ethical clearance by the College Research Ethics Committee at King's College London (MRA-21/22-28555).

### Participants

Participants were recruited via both purposive and snowballing strategies: individuals were initially approached due to their eligibility and suitability, and were simultaneously provided with the participant eligibility criteria and encouraged to approach their colleagues and networks to seek further study participants. A two-page invitation document was produced in English, French, Spanish, and Arabic. The document contained (1) a summary of the study context, objectives, and methods; (2) a weblink to the Research Electronic Data Capture (REDCap) webpage for individuals wishing to participate; and (3) a description of our target participants, alongside an invitation to share the document with potentially suitable colleagues.

A database was manually created to distribute the study invitation, using online search engines and research databases to compile contact details for eligible individuals. These searches aimed to identify up to four suitable individuals from each one of all 138 LMICs as defined by the World Bank.^30^ We sought to identify clinicians or researchers who (1) appeared to have more than 10 years' experience working in the cancer field; (2) preferably lived in an RCR (although individuals living in HICs with an extensive record of working in the RCR context were considered); and (3) who appeared to have an academic presence in the form of multiple peer-reviewed research publications. Efforts were made to include, from each country, (1) both clinicians and researchers; (2) both those specializing in adult cancers, and those specializing in pediatric cancers.

Individuals identified as suitable and contactable were sent a personalized e-mail sent from the e-mail address of the first author (L.F.), with L.F., M.V.H., R.S., D.M., V.V., and L.M.-H. as signatories, and with the invitation document attached. Those contacted were sent a reminder e-mail 2 weeks after the initial invitation.

### Procedures

Individuals who followed the link to the web survey and provided their informed consent to participate were directed to an online questionnaire powered by REDCap with four language options: English, French, Spanish, and Arabic. Translations from English were produced using AI-powered language translation tools, then validated for accuracy and comprehension with the assistance of collaborators fluent in the target language. To inform their responses, all participants were provided with previously published work from REPRISE that documented the impacts of the COVID-19 pandemic on the cancer research field.^19,31,32^

#### Round 1

In round 1, the participant was asked questions about their country of residence and professional background. Then, they were presented with two sections:

1. A list of 25 predefined *general cancer* ARPs produced by previously published collaborative work within our Cancer and COVID-19 Global Task Force^28^ (section 1).
2. A further seven *COVID-19/cancer* ARPs produced by the authors for this study (section 2).

The participant was asked to rate each ARP on a scale from 1 (of limited priority) to 9 (of utmost priority). After each section, the participant was provided with a free-text box and asked if they thought any ARP was missing from the list of ARPs provided in that section (Fig 2).

After closing round 1, we reformulated the text provided within the free-text boxes for comprehension, into statements which formed additional ARPs. To do this, the first author (L.F.) reformulated the text into provisional statements, which were then reviewed by three other authors (D.M., J.T., and M.V.H.), each alongside the original text provided by the participant. The process resulted in minor revisions to the proposed statements and merging of some, until all four authors agreed on all statements. The resulting 43 ARPs (29 new general cancer ARPs and 14 new COVID-19/cancer ARPs) were taken to round 2.

#### Round 2

As in round 1, the round 2 questionnaire consisted of two sections: general cancer ARPs (section 1); and COVID-19/cancer ARPs (section 2; Fig 2). In each section, the participant was asked to provide a rating from 1 to 9 for each new ARP (as in round 1). Then, in each section, they were presented with a combined list of all ARPs—that is, the predefined ARPs and the new participant-derived ARPs. The combined lists numbered 54 general cancer ARPs in section 1; and 21 COVID-19/cancer ARPs in section 2. In section 1, the participant was compelled to choose, in their view, on the basis of their locoregional experience, their top 10 ARPs, then their top five ARPs, then their top one ARP. In section 2, considering the smaller list size, the participant was asked to select their top four ARPs, then their top one ARP.

#### Validation Exercise

The validation exercise with patient organization representatives took the form of three 1-hour 1:1 semistructured interviews with three interviewees who hold respective leadership roles in the *World Bladder Cancer Patient Coalition*, *World Ovarian Cancer Coalition*, and *Advanced Breast Cancer Global Alliance*. In the interviews conducted by L.F. (an experienced qualitative and patient-reported outcome measures researcher), the study findings were scrutinized item-by-item, so that the interview participants could comment both on individual ARPs and contribute more generalized comments. L.F. took handwritten notes of all interviewee contributions, and all notes were read back to the interviewee at the end of the interview to mitigate against misinterpretation or misrepresentation.

### Analysis

**FIG 3:**
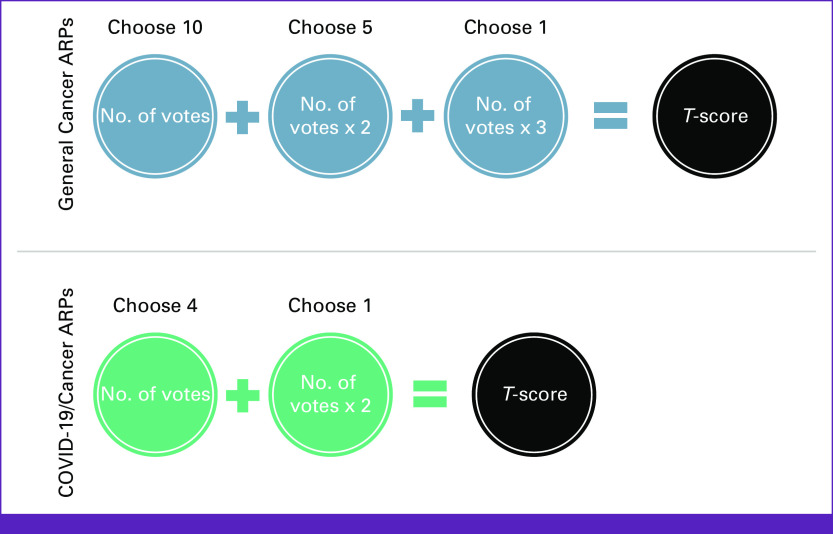
Scoring equation for total composite scores (*T*-scores) for general cancer and COVID-19/cancer ARPs. ARPs, areas of potential research priority.

Data collected from round 2 formed the basis of the study analysis. For participants' ratings of the importance of each individual ARP (ie, between 1 and 9), responses were analyzed descriptively, and measures of central tendency (mean) and dispersal (standard deviation [SD]) were produced. For the ranking exercise (ie, the selection of highest priorities), the number of votes was counted for each ARP, for each stage of the ranking exercise. Thus, for each general cancer ARP, it was counted how many respondents selected that ARP as one of the top 10 priorities; how many selected it as one of the top five; and how many selected it as the single greatest priority, respectively. For each COVID-19/cancer ARP, it was counted how many participants selected it as their top four; and as the single greatest priority, respectively. That counting process produced three basic vote totals for general cancer ARPs and two basic vote totals for COVID-19/cancer ARPs. Then, the ARP scores were weighted and summed into a *total composite score* (*T*) as shown in Figure 3.

The weighting and summing procedures in Figure 3 were agreed by L.F., A.S. and M.V.H., upon following a cursory preliminary analysis, which appeared to demonstrate a degree of reliability and consistency in the data regarding the popularity of particular ARPs across the different stages of ranking.

The analyses were conducted using SAS statistical software v9.4 (SAS Institute Inc, Cary, NC). Study results are presented below as the ARP list ranked by *T* scores, alongside basic vote totals and mean importance ratings of each ARP with SDs.

Handwritten notes produced during the validation exercise were subject to basic content analysis to distil all salient contributions made by the patient organization representatives. This process was selective only in the sense that superfluous or irrelevant notes taken during the natural flow of the conversation were disregarded; the analysis aimed to comprehensively include all relevant contributions made by the interviewees without researcher discretion.

## RESULTS

We were able to find eligible individuals with available contact details from 97 LMICs, comprising 157 individuals in total (including 13 members of our Task Force). All 157 individuals were contacted. Six invitations were flagged as undeliverable by the e-mail server, resulting in 151 individuals successfully being contacted. The global distribution of these 151 individuals by WHO regions is shown in Table 1.

**TABLE 1:**
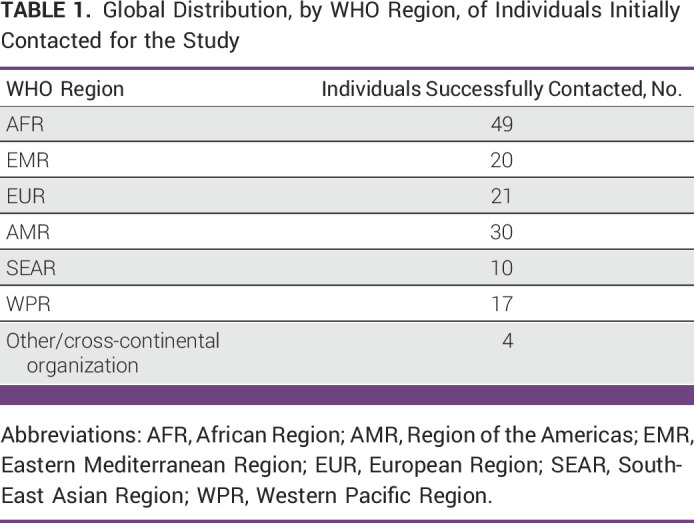
Global Distribution, by WHO Region, of Individuals Initially Contacted for the Study

**FIG 4:**
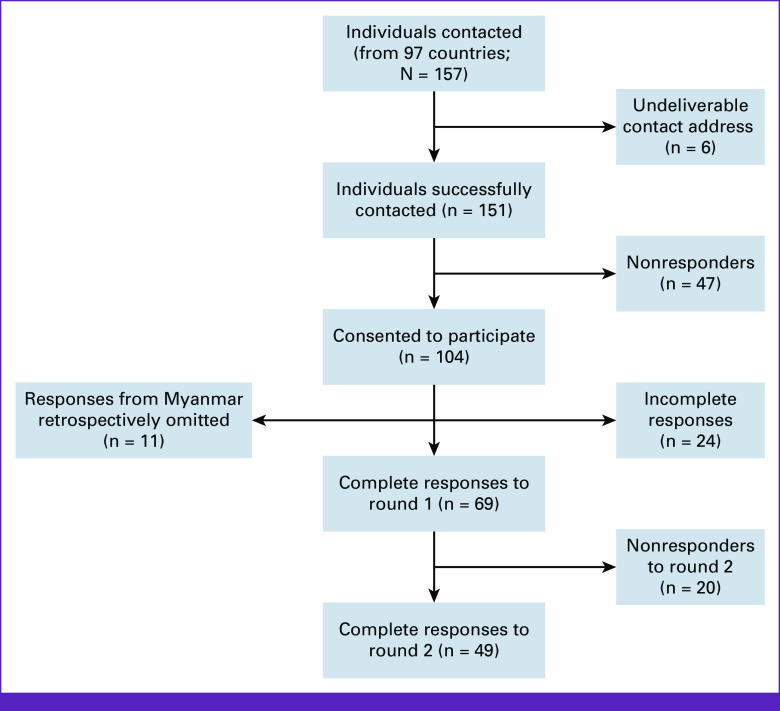
Flowchart of participant inclusion.

Of the 151 individuals contacted, 104 unique individuals participated in round 1 of the survey. Of these, 24 did not complete the questionnaires fully, resulting in n = 80 complete individual responses in round 1. In round 2, n = 60 of those 80 individuals participated (ie, n = 20 nonresponders). Of the participants in round 2, n = 7 did not respond to the COVID-19–related section of round 2 (only the first section on general cancer research). Upon examination of the responses, it was apparent that individuals residing in Myanmar were strongly over-represented in the data (n = 13 [16% of the sample] in round 1; n = 13 [22% of the sample] in round 2). A sensitivity analysis was conducted with the Myanmar participants omitted, from which it was judged that the inclusion of these participants substantively skewed the results. Hence, in the interest of analytical balance, n = 2 participants from Myanmar (ie, consistent with the mean number of responses from other countries) were randomly selected using a computer and only those participants were included in the analysis; the remaining 11 participants from Myanmar were omitted. Therefore, the final sample comprised n = 69 individuals in round 1, and n = 49 individuals in round 2 (Fig 4). The characteristics of this sample are shown in Table 2.

**TABLE 2:**
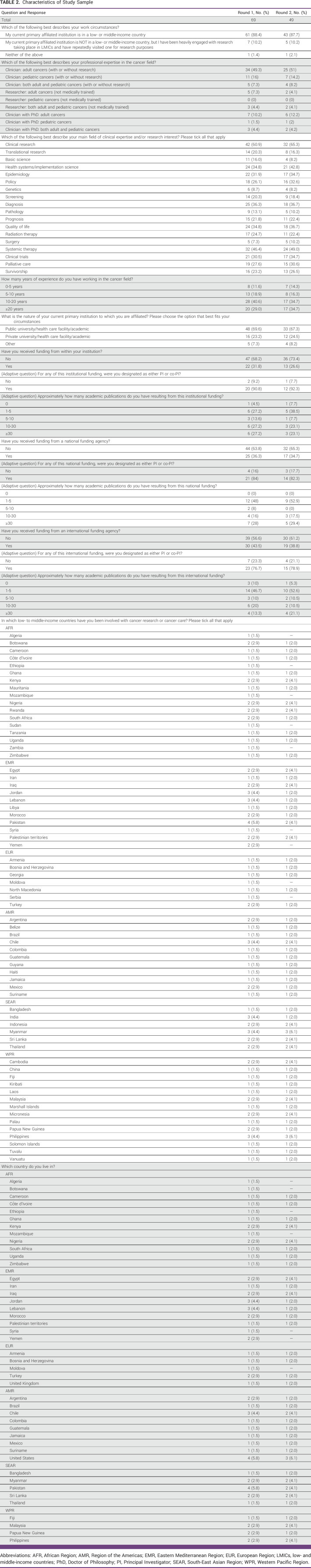
Characteristics of Study Sample

Final ARP score data from round 2 are shown in Table 3 (general cancer ARPs) and Table 4 (COVID-19/cancer ARPs). Composite voting scores (ie, *T* scores) are shown alongside mean values and SDs for the 1-9 ratings of each individual ARP.

**TABLE 3:**
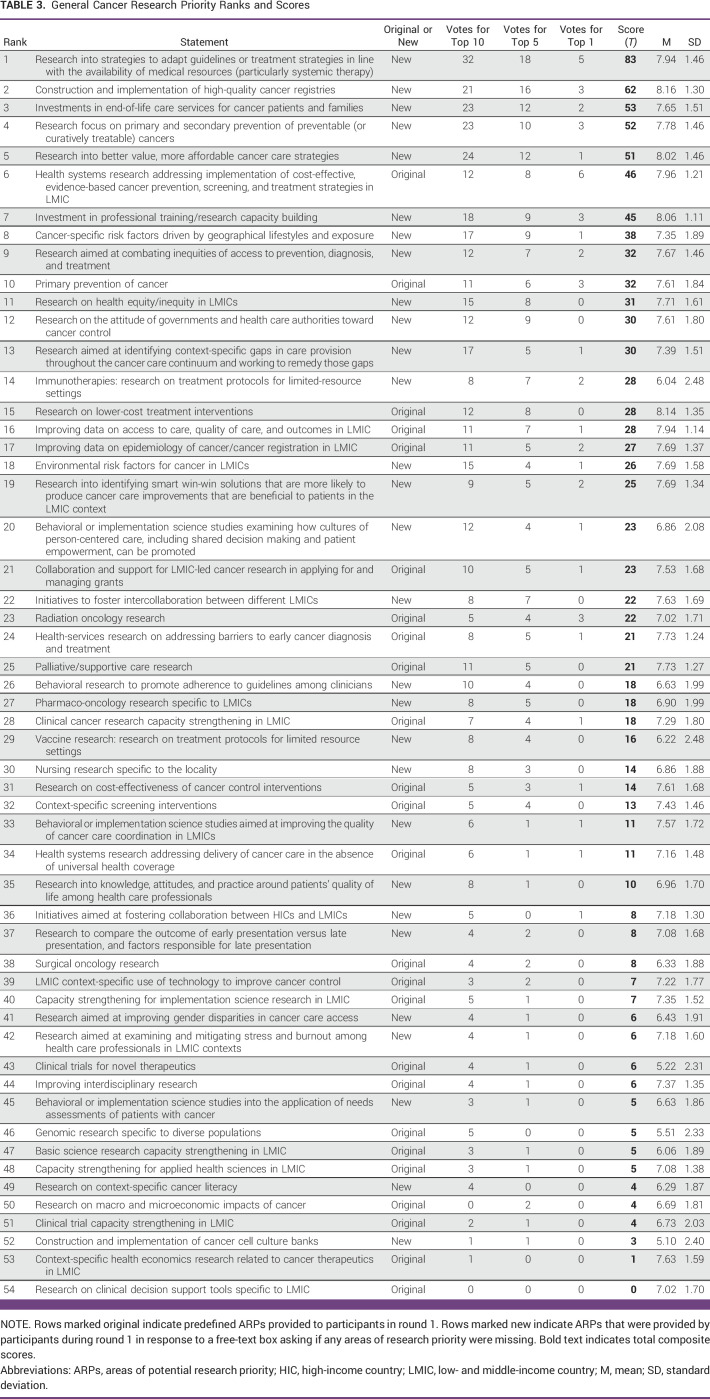
General Cancer Research Priority Ranks and Scores

**TABLE 4:**
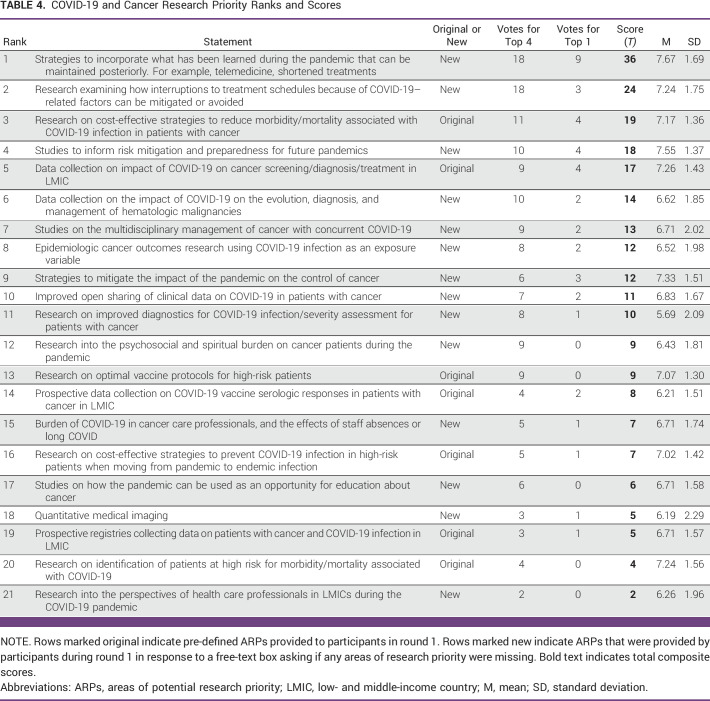
COVID-19 and Cancer Research Priority Ranks and Scores

The top five general cancer ARPs, according to their *T* score, were:

1. Research into strategies to adapt guidelines or treatment strategies in line with the availability of medical resources (particularly systemic therapy; *T* = 83).
2. Construction and implementation of high-quality cancer registries (*T* = 62).
3. Investments in end-of-life care services for cancer patients and families (*T* = 53).
4. Research focus on primary and secondary prevention of preventable (or curatively treatable) cancers (*T* = 52).
5. Research into better value, more affordable cancer care strategies (*T* = 51).

The top five COVID-19/cancer ARPs, according to their *T* score, were:

1. Strategies to incorporate what has been learned during the pandemic that can be maintained posteriorly. For example, telemedicine, shortened treatments (*T* = 36).
2. Research examining how interruptions to treatment schedules because of COVID-19–related factors can be mitigated or avoided (*T* = 24).
3. Research on cost-effective strategies to reduce morbidity/mortality associated with COVID-19 infection in patients with cancer (*T* = 19).
4. Studies to inform risk mitigation and preparedness for future pandemics (*T* = 18).
5. Data collection on the impact of COVID-19 on cancer screening/diagnosis/treatment in RCRs (*T* = 17).

The interviews with representatives of patient advocacy organizations produced the following feedback:

1.General agreement that the top set of priorities identified deserves to be prioritized from their perspective.
2.Generally speaking, the findings are consistent with their experiences and previous work.
3.Agreement that the top priority identified in each category is highly important.

Upon reviewing all study results, some specific issues were raised by the patient organization representatives (Table 5).

**TABLE 5:**
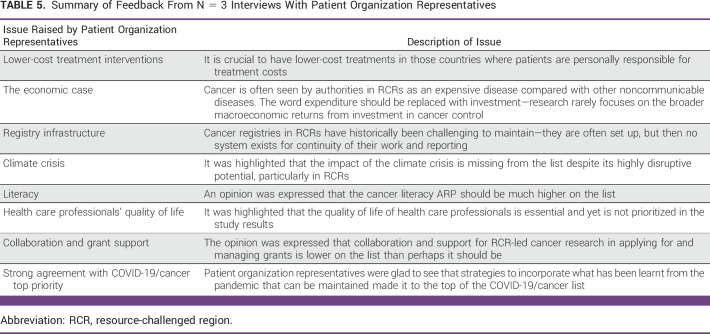
Summary of Feedback From N = 3 Interviews With Patient Organization Representatives

## DISCUSSION

The findings of this study, which aimed to center the voices of experts in RCRs in the study procedures, showed that there are some clear areas of priority for cancer research in RCRs that many of those experts can agree on. In summary, these relate primarily to (1) adapting treatment guidelines to suit resource-limited settings (particularly systemic therapy); (2) sustainable implementation of cancer registries; (3) prevention strategies; (4) value-based and affordable cancer care; (5) health systems research focused on the implementation of value-based care; (6) investments in research capacity building; (7) epidemiologic work on local risk factors for cancer; and (8) combatting inequities of prevention and care access.

Our validation work also noted that environmental shocks, health literacy, health care workers' quality of life, and support for RCR-led research in applying for and managing grants were key to this conversation. From a more strategic perspective, it was noted that a valid economic case should more often be made for cancer control as a macroeconomic investment in health systems and public health, rather than an expenditure.

In terms of COVID-19 response, the areas of priority in our study relate primarily to (1) taking forward and comprehensively investigating alternative care strategies, such as telemedicine, which were used during the pandemic; (2) examination of how treatment interruptions can be avoided during a disease outbreak akin to COVID-19; (3) strategies on how to reduce COVID-19–related morbidity in those with cancer; (4) preparing for future pandemics with risk mitigation strategies; and (5) analyzing the impact of COVID-19 on cancer control.

To aid the interpretation of the above findings, we presented our results at the World Cancer Congress, Geneva, on October 18, 2022. We discussed our observations with cancer experts from RCRs and global cancer control experts, who raised these points of interpretation:

*Armed conflict* was highlighted as part of a wider discussion about external factors such as regional instability, which also relates to climate and environmental crises. There is a need to consider national and geospatial variation in these contexts; and questioning of the broad designation of the term LMIC, given regional heterogeneity in these and other regards.

*Adaptive treatment guidelines* were discussed, particularly the potential need for an informed update of adaptive guidelines, considering that treatment protocols are ever-changing, which is likely to require additional study.

*Oncology nurses* were suggested as needing a stronger voice in the conversation about research priorities.

*Research engagement in RCRs* (or lack thereof) was identified as a barrier to a thriving local research ecosystem. A contributor highlighted the preconceptions about research among certain communities. How can the benefits of research be promoted?

Finally, *engagement with policymakers* was evaluated as a key issue: there is a gap between the available published research and the amount of research publications cited in policy documents.

In the context of these last two points, it also needs to be noted that despite an ever-increasing majority of the global cancer burden residing in RCRs, there appears to be a funding and publication bias against RCTs conducted in RCRs—although trials in RCRs are more likely than HIC trials to identify effective therapies for large cohorts and produce greater effect sizes.^33^ In an important analysis of all phase III oncology RCTs between 2014 and 2017, Wells et al^33^ have laid out this imbalance and observed that “the global research agenda is dominated by the pharmaceutical industry in HICs … there is an urgent need for philanthropic and government funding agencies to recalibrate this imbalance.”^(p382)^

To our knowledge, our study is thus the first to collate the views of individuals working in RCRs about cancer research priorities in the wake of the COVID-19 pandemic. A strength of our study is the global reach of the sample, which is considerable. However, it should be noted that the scope of this study is confined to generalized information about RCRs and is not necessarily reflective of the specific contexts of individual regions and nations. It is also possible that the findings are more representative of the views of oncologists and researchers than of the views of nurses and patients.

To conclude, to our knowledge, this is the first globally representative exercise to establish cancer research priorities for RCRs after COVID-19. The work we have undertaken for REPRISE shows the importance of diverse teams, and context-specific research questions driven by researchers and patients on the ground. Furthermore, we wish to call for an evolution of global facing funding frameworks for cancer research, to be fit for purpose in a new era of building resilient cancer systems.
